# Transperineal ultrasonography in detecting penetrating perianal disease: a systematic review and meta-analysis

**DOI:** 10.1093/ecco-jcc/jjag032

**Published:** 2026-03-24

**Authors:** Chong-Teik Lim, Maarten Pruijt, Gek-Hsiang Lim, Faridi Jamaludin, Christoph Teichert, Floris de Voogd, Geert D’Haens, Britt Christensen, Giovanni Maconi, Krisztina Gecse

**Affiliations:** Department of Gastroenterology and Hepatology, Amsterdam University Medical Centre, Amsterdam, 1081 HV, The Netherlands; Department of Gastroenterology and Hepatology, Singapore General Hospital, 169856, Singapore; Department of Gastroenterology and Hepatology, Amsterdam University Medical Centre, Amsterdam, 1081 HV, The Netherlands; Health Services Research Unit, Singapore General Hospital, 169608, Singapore; Medical Library AMC, Amsterdam UMC location University of Amsterdam, Amsterdam, 1105 AZ, The Netherlands; Department of Gastroenterology and Hepatology, Amsterdam University Medical Centre, Amsterdam, 1081 HV, The Netherlands; Department of Gastroenterology and Hepatology, Amsterdam University Medical Centre, Amsterdam, 1081 HV, The Netherlands; Department of Gastroenterology and Hepatology, Amsterdam University Medical Centre, Amsterdam, 1081 HV, The Netherlands; Department of Gastroenterology, The Royal Melbourne Hospital, Parkville,VIC 3050, Australia; Department of Medicine, University of Melbourne, Parkville, VIC 3050, Australia; Gastroenterology Unit, Department of Biomedical and Clinical Sciences, ‘L. Sacco’ Hospital, University of Milano, Milan 20157, Italy; Department of Gastroenterology and Hepatology, Amsterdam University Medical Centre, Amsterdam, 1081 HV, The Netherlands

**Keywords:** transperineal ultrasound, TPUS, perianal fistula, abscess, perianal fistulizing Crohn’s disease

## Abstract

**Background and Aims:**

Perianal complications such as fistulas and abscesses are common in Crohn’s disease (CD) and contribute to significant morbidity. Transperineal ultrasonography (TPUS) has emerged as a non-invasive and accurate method for perianal fistulizing CD (pfCD). This review evaluates the diagnostic accuracy of TPUS compared with magnetic resonance imaging (MRI), transrectal ultrasonography (TRUS), and examination under anesthesia (EUA) for detecting and classifying perianal fistulas and abscesses.

**Methods:**

A comprehensive literature search was conducted across multiple databases through January 2025 to identify studies evaluating TPUS accuracy in detecting perianal fistulas and abscesses compared with MRI, TRUS, or EUA as the reference standard. Meta-analysis was performed to assess TPUS accuracy for fistula detection (FD), fistula classification (FC), internal opening (IO) detection, and abscess detection (AD). The Quality Assessment of Diagnostic Accuracy Studies-2 tool was used to evaluate risk of bias.

**Results:**

Of 1059 studies identified, 29 were included in this review. Pooled sensitivities for FD (18 studies, 1474 patients), FC (11 studies, 585 patients), IO detection (six studies, 481 patients), and AD (16 studies, 1276 patients) were 97.5%, 80.3%, 89.6%, and 93.5% respectively while pooled specificities for FD, IO detection, and AD were 69.0%, 66.3%, and 94.5% respectively. The overall TPUS accuracy for FD, FC, IO detection, and AD was 88.0%, 88.6%, 77.8%, and 91.8% respectively. Subgroup analysis on CD patients showed an accuracy of 86.4%, 87.6%, and 83.3% for FD, FC, and AD respectively.

**Conclusions:**

TPUS demonstrates high accuracy in detecting perianal fistulas and abscesses, supporting its use as a non-invasive, first-line diagnostic tool.

## 1. Introduction

Crohn’s disease (CD) is a chronic, immune-mediated inflammatory disorder characterized by transmural inflammation affecting any location along the gastrointestinal tract.^1^ Perianal fistulizing CD (pfCD) contributes to considerable morbidity and poor disease prognosis. Perianal fistulizing CD can arise concurrently with, after, or even before the diagnosis of luminal CD.^2^ Accurate diagnosis and classification of pfCD are crucial for implementing timely and effective treatment.^3^

Multiple diagnostic methods exist for pfCD including examination under anesthesia (EUA), transrectal endoscopic ultrasonography (TRUS), magnetic resonance imaging (MRI), and transperineal ultrasonography (TPUS). Currently MRI is considered the gold standard for diagnosing pfCD, which provides detailed images of deep tissues and superior soft tissue differentiation, enabling comprehensive assessment of fistula tracts relative to perianal anatomy.^4^ However, MRI is associated with high cost, lengthy scan times, and waiting lists, which all make it less practical for patients requiring frequent evaluations to monitor pfCD progression or healing. TPUS, by contrast, offers a non-invasive, reliable, patient-friendly, and safe alternative for evaluating pfCD.^5^ It requires no special preparation and provides real-time images, making it well-suited for repeated assessments that also enable immediate adjustment of therapy.

Since the last comprehensive review in 2017,^6^ ultrasound imaging—particularly transperineal and transabdominal techniques—has seen notable advancements in inflammatory bowel disease (IBD), resulting in a significant increase in published studies. Additionally, improved image resolution in ultrasound technology has enhanced diagnostic precision.^7^ This systematic review and meta-analysis aim to provide an updated assessment of the diagnostic accuracy of TPUS in comparison to MRI, TRUS, and EUA for evaluating perianal manifestations of CD with a specific focus on detecting and classifying perianal fistulas and abscesses.

## 2. Methods

This systematic review was conducted in accordance with the Preferred Reporting Items for Systematic Reviews and Meta-Analyses of Diagnostic Test Accuracy Studies (PRISMA-DTA) guidelines.^8^ The review protocol was registered on PROSPERO under ID CRD 42024511822 and is accessible at PROSPERO (Supplementary Files 1).

### 2.1. Search strategy

The comprehensive literature search was conducted with the assistance of a scientific librarian from the Amsterdam University Medical Center (F.S.) utilizing MEDLINE [Ovid] and EMBASE [Ovid] databases, covering publications from inception to February 4, 2025. The search strategy included terms [“transperineal” OR “perineal” OR “perianal”] AND [“fistula” OR “abscess”] AND [“ultrasonography” OR “ultrasono-” OR “-sonography”]. Full search details and results are available in Supplementary Files 2.

### 2.2. Study eligibility criteria

Studies were included if they involved patients with a confirmed or suspected perianal fistula or abscess and TPUS was used in conjunction with at least one other modality of TRUS, MRI, and/or EUA. All studies meeting these criteria were included regardless of publication status or language. Case series or studies with small sample size (fewer than 10 patients) were excluded. For studies where the full article was unavailable, we contacted primary authors by email to request the complete text.

Two reviewers (C.T.L. and M.P.) independently screened study titles and abstracts, followed by full text assessment for eligibility. Any discrepancy was first resolved by consensus or, if required, by a third reviewer (K.G.). We also reviewed the references of previously published meta-analysis,^6^ reviews, and eligible articles to identify additional studies.

### 2.3. Data extraction

Data regarding study characteristics (title, authors, authors’ country, publication year, study design, and sample size), clinical characteristics (patients’ age, gender, and presence of CD), TPUS data and techniques (TPUS operator’s medical specialty, ultrasound system, transducer probe used, mode [B-mode, color Doppler and/or Power Doppler], use of contrast to enhance fistula tracts [hydrogen peroxide, SonoVue, and/or normal saline injection], and additional techniques described) and reference standard interpretation (types of confirmatory investigation performed, blinding for ultrasonographic findings, and time interval between TPUS to reference standard) were extracted by one reviewer (C.T.L.) and verified by a second reviewer (M.P.).

### 2.4. Outcomes

The primary outcome of this meta-analysis was to assess the diagnostic accuracy of TPUS in detecting perianal fistulas (FD) compared to TRUS, MRI, and/or EUA. Secondary outcomes included evaluating TPUS accuracy in classifying perianal fistulas (FC), identifying internal fistula openings (IO), and detecting perianal abscesses (AD).

### 2.5. Methodological quality

The methodological quality and risk of bias of each study were evaluated using the Quality Assessment of Diagnostic Accuracy Studies (QUADAS-2) tool.^9^ The QUADAS-2 tool consists of a series of questionnaires for each domain to assess quality with risk of bias classified as “low,” “high,” or “unclear” depending on the answer to the signaling questions. Quality assessment was performed by two reviewers (C.T.L. and M.P.) with any discrepancy resolved by consensus or by a third reviewer (K.G.) if required.

### 2.6. Data analysis

Data from eligible studies were analyzed using a bivariate random effects model. For all domains (FD, FC, IO, and AD), there were a few studies with no false positives or true negatives that did not permit specificity calculations. Studies with sparse data (defined as 100% sensitivity and/or 100% specificity resulting in zero-cell counts) were excluded from bivariate random effects meta-analysis because such data precluded estimation of sensitivity–specificity correlation. However, these studies were included in sensitivity analyses, where pooled sensitivity and specificity were estimated using a fixed-effects logistic regression model.^10^ Where more than one reference standard (MRI, EUA, and/or TRUS) was used within a study, these were treated as a composite reference standard in the primary analysis and weighted equally. Results were visualized using forest plots and summary receiver operating characteristics curves.

Analyses were conducted using Stata 17 (StataCorp, College Station, TX, USA). Specifically, the *metadta*, *metandi*, *midas*, and *blogit* functions were used.

## 3. Results

### 3.1. Study inclusion

**Figure 1.**
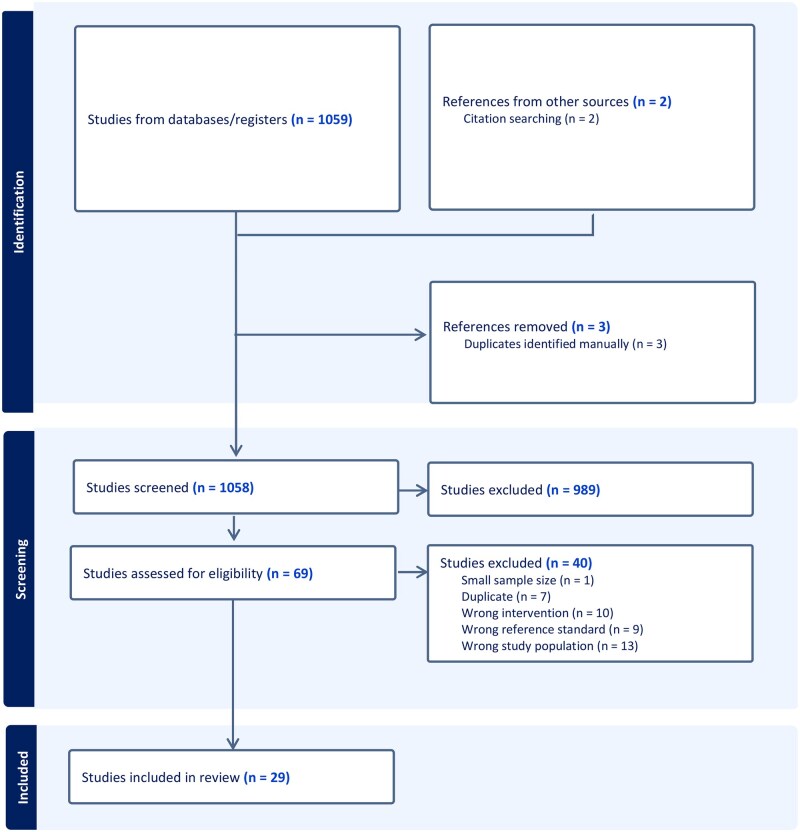
PRISMA (Preferred Reporting Items for Systematic Reviews and Meta-Analyses) flowchart.

From a total of 1059 articles identified through the search strategy, 989 were excluded after screening titles and abstracts (Figure 1). The full texts of the remaining 69 studies were retrieved, of which 29 studies met inclusion criteria for systematic review and meta-analysis. Reasons for exclusion included incorrect patient population (*n* = 13), non-applicable intervention (*n* = 10), lack of an appropriate reference standard (*n* = 9), duplicates (*n* = 7), and sample sizes under 10 patients (*n* = 1).

### 3.2. Risk of bias and concerns of applicability

Overall, the studies demonstrated a moderate risk of bias, with moderate applicability concerns (Table S1 and Figure 1). Of the 29 included studies,^11–39^ only five presented low risk of bias while nine had low applicability concerns. Patient selection and reference standard were the two key areas with high risk of bias. This was due to non-random sampling or non-consecutive enrolment in patient selection and insufficient reporting on IBD patients for applicability. Additionally, a moderate to high risk of bias was noted in the reference standard domain, due primarily to unblinded interpretations of the reference standard results.

### 3.3. Study characteristics

Table 1 summarizes the included studies. The aims of the studies include FD (*n* = 27), FC (*n* = 13), AD (*n* = 18), and IO detection (*n* = 14). Nineteen studies (65.5%)^11–15^^,^^17^^,^^18^^,^^21–25^^,^^31^^,^^32^^,^^35–38^ had a prospective design, with sample sizes ranging from 13 to 492 and a combined total of 2023 patients. Most participants (74.0%) were male. Thirteen studies^11^^,^^13^^,^^14^^,^^16–19^^,^^21–23^^,^^26^^,^^30^^,^^34^ reported IBD patient numbers, though only 10 studies^11^^,^^14^^,^^17–19^^,^^21–23^^,^^26^^,^^30^ (410 patients) provided sufficient data for analysis.

**Table 1.** Characteristics of studies included.

First author	Year	Design	Country	Language	Total number of participants	Gender (male)	Number of IBD patients	Age, years (mean unless stated otherwise)	Aims
**Stewart** ^11^	2001	Prospective	Canada	English	54	28	31	Range (23-69)	FD, FC
**Bonatti** ^12^	2004	Prospective	Germany	German (English abstract)	44	34	–	49	FD
**Mallouhi** ^13^	2004	Prospective	Austria	English	87	54	22	45 ± 15	FD, AD
**Wedemeyer** ^14^	2004	Prospective	Germany	English	25	8	25	36.2 ± 2.5	FD, AD
**Zbar** ^15^	2006	Prospective	Barbados	English	20	15	–	45.5 Range (28-82)	FD, FC, AD
**Domkundwar** ^16^	2007	Retrospective	India	English	30	26	1	37.9 Range (24-56)	FD, IO
**Kleinubing** ^35^	2007	Prospective	Brazil	English	43	32	–	39 Range (18-76)	FD, IO
**Maconi** ^17^	2007	Prospective	Italy	English	46	21	46	Median: 37 Range (16-72)	FD, FC, AD
**Maconi** ^18^	2013	Prospective	Italy	English	59	27	59	39 Range (19-72)	FD, FC, AD
**Nevler** ^19^	2013	Retrospective	Israel	English	41	28	14	Median a. CD: 28 b. non-CD: 40	FD, FC, IO, AD
**Plaikner** ^20^	2014	Retrospective	Austria	English	67	40	–	Median: 44 (IQR: 35-50)	FD, AD
**Terracciano** ^21^	2014	Prospective (Abstract)	Italy	English	13	7	13	11	FD
**Bor** ^23^	2016	Prospective	Hungary	English	23	11	23	29.9	FD, FC, AD
**Terracciano** ^22^	2016	Prospective	Italy	English	28	17	28	37.6 ± 16	FD, FC, AD
**Puranik** ^24^	2017	Prospective	India	English	492	432	–	Range (17-89)	FD, AD
**Fateh** ^25^	2017	Prospective	Iraq	English	51	42	–	37.2 Range (15-67)	FD, FC, IO, AD
**Lee** ^26^	2018	Retrospective	South Korea	English	38	26	38	14.7 Range (5.8-19.6)	FD, AD
**Yan** ^27^	2018	Retrospective	China	Chinese (English abstract)	36	36	–	39.9 ± 13.9	FD, IO
**Anand** ^28^	2022	Retrospective	India	English	74	44	–	NA	FD, AD
**Ding** ^29^	2022	Retrospective	China	English	203	203	–	Range (0-3)	FC
**Jung** ^30^	2022	Retrospective	South Korea	English	125	19	125	Median 14 Range (8-18)	FD
**Boles** ^31^	2022	Prospective	Egypt	English	30	25	–	43.5 Range (20-68)	FD, IO, AD
**Singh** ^32^	2022	Prospective	India	English	37	32	–	Range (45-60)	FD, IO, AD
**Altam** ^33^	2023	Retrospective	Yemen	English	85	69	–	32.12 ± 13.83	FD, FC, IO, AD
**Hosokawa** ^34^	2023	Retrospective	Japan	English	52	37	27	6.7 ± 6.4	FD, AD
**Garg** ^36^	2023	Prospective	India	English	50	43	–	43.3 Range (30-60)	FD, FC, IO
**Yang** ^37^	2024	Prospective	China	English	60	42	–	37.1 ± 11.4 Range (20-72)	FC, IO
**Islam** ^38^	2024	Prospective	Bangladesh	English	50	43	–	Range (21-60)	FD, IO
**Chang** ^39^	2025	Retrospective	China	English	60	57	–	38.5 Range (20-65)	FD, FC, IO, AD

Abbreviations: FD, fistula detection; FC, fistula classification; IO, internal opening; AD, abscess detection; NA, not applicable.

Table 2a summarizes the characteristics of TPUS across studies. The TPUS operators were specified in 22 studies^11–20^^,^^22^^,^^25^^,^^26^^,^^28–32^^,^^34–36^^,^^39^ and were primarily radiologists (*n* = 15) followed by gastroenterologists (*n* = 4) and surgeons (*n* = 3). The reference standards varied and included EUA, TRUS, and cross-sectional imaging (MRI or computed tomography [CT]) with MRI usage increasing after 2012. There were 14 studies^14^^,^^15^^,^^17–20^^,^^22^^,^^26^^,^^30^^,^^31^^,^^34^^,^^36^^,^^37^^,^^39^ (48.3%) of which the TPUS operators were blinded to the reference standard. Time interval between reference standard and TPUS varied widely and were reported in 16 studies^13–15^^,^^17–23^^,^^25^^,^^26^^,^^30^^,^^31^^,^^33^^,^^37^ (55.2%).

**Table 2a.** Characteristics of TPUS studies performed.

Study	Year	TPUS operator	Blinding for RS	US system	Transducer Probe	RS	Time interval RS and TPUS
**Stewart** ^11^	2001	Radiologist	NA	ATL US (USA)	Linear (7-12 MHz) Transvaginal (8-14 MHz)	EUA	NA
**Bonatti** ^12^	2004	Radiologist	NA	NA	Linear (7 MHz) Sector (3.5 MHz)	MRI, CT, EUA	NA
**Mallouhi** ^13^	2004	Radiologist	NA	HDI 5000 (Philips, USA)	Linear (4-7 MHz)	EUA	Mean 2.4 ± 3 days
**Wedemeyer** ^14^	2004	Gastroenterologist	Yes	Aplio, Powervision (Toshiba, Japan) or Elegra (Siemens, Germany)	Linear (3.3-12 MHz)	MRI	Median 10 days (range: 0-75)
**Zbar** ^15^	2006	Surgeon	Yes	B-K (Denmark)	Curvilinear (7.5 MHz)	TRUS, **EUA**	<28 days
**Domkundwar** ^16^	2007	Surgeon	NA	Eccocee, Justvision, or Nemio (Toshiba, Japan)	Linear (7-11 MHz) Sector (3-6 MHz) Transvaginal (5-7 MHz)	EUA	NA
**Kleinubing** ^35^	2007	Surgeon	NA	Diasonic Logic TM 400 (GE, USA)	Linear (7-10 MHz) Transvaginal (5-7.5 MHz)	EUA	NA
**Maconi** ^17^	2007	Gastroenterologist	Yes	NA	Linear (7.5 MHz) Convex (3.5-5 MHz)	TRUS	Same day
**Maconi** ^18^	2013	Gastroenterologist	Yes	NA	Microconvex (4-8 MHz)	MRI, EUA	Mean 10 days
**Nevler** ^19^	2013	Gastroenterologist	Yes	B-K (Denmark)	Curvilinear (7.5 MHz)	EUA	Mean 35 days
**Plaikner** ^20^	2014	Radiologist	Yes	HDI 5000 (Philips, USA)	Linear (5-7 MHz) Convex (5-8 MHz)	MRI, **EUA**	Mean 8 days (range 0-58)
**Terracciano** ^21^	2014	NA	NA	NA	Mini Convex (4-7 MHz)	MRI, EUA	Mean 20 days
**Bor** ^23^	2016	NA	NA	NA	Microconvex (16-36 MHz)	EUA	Mean 7 days (range 0-29)
**Terracciano** ^22^	2016	Radiologist	Yes	NA	Microconvex (4-8 MHz)	MRI	Mean 11.4 ± 9.4 days
**Puranik** ^24^	2017	NA	NA	Logiq 5 (GE, USA)	Linear (7-13 MHz) Sector (2-5 MHz)	Clinical, EUA, MRI	NA
**Fateh** ^25^	2017	Radiologist	No	Medison 2.00 Sonoace X8	Linear (7-12 MHz)	MRI	Mean 25.2 days (range 7–70)
**Lee** ^26^	2018	Radiologist	Yes	iU22 (Philips, USA)	Linear (5-12 MHz)	MRI	Mean 4.6 ± 11.9 days
**Yan** ^27^	2018	NA	NA	iU22, iU Elite or Epiq5(Philips, USA)	Linear (5-12 MHz) Convex (3-10 MHz)	EUA	NA
**Anand** ^28^	2022	Radiologist	NA	NA	Linear (3-8 MHz) Sector (2-5 MHz)	MRI	NA
**Ding** ^29^	2022	Radiologist	NA	Mylab Twice (Esaote, Italy)	Linear (18 MHz)	EUA	NA
**Jung** ^30^	2022	Radiologist	Yes	iU22 or Epiq7 (Philips, USA)	Linear (5-12 MHz)	MRI	<14 days
**Boles** ^31^	2022	Radiologist	Yes	Aplio 500 (Toshiba, Japan)	Linear (5-12 MHz) Curvilinear (5-8 MHz)	MRI	<14 days
**Singh** ^32^	2022	Radiologist	NA	Voluson E8 (Wipro GE, India)	Linear (3-8 MHz) Sector (2-5 MHz)	MRI	NA
**Altam** ^33^	2023	NA	NA	WS850 (Samsung, South Korea)	Curve (4-7 MHz)	EUA	<10 days
**Hosokawa** ^34^	2023	Radiologist	Yes	Logiq7, E9, S8 or E10 (GE, USA)	Linear (9-15 MHz)	CT, MRI	NA
**Garg** ^36^	2023	Radiologist	Yes	GE VOLUSON S6	Linear (7-12 MHz) Curvilinear (2-5 MHz)	MRI	NA
**Yang** ^37^	2024	NA	Yes	GE Voluson E10 diagnostic ultrasound system	180 degrees rotating 3D volume probe (frequency 5-9 MHz) was used	EUA	<24 h
**Islam** ^38^	2024	NA	NA	NA	NA	EUA	NA
**Chang** ^39^	2025	Radiologist	Yes	GE LOGIQ 5	Linear (12 MHz)	EUA	NA

Abbreviations: TPUS, transperineal ultrasound; RS, reference standard; NA, information not available; TRUS, transrectal ultrasonography; CT, computed tomography; MRI, magnetic resonance imaging; EUA, examination under anesthesia. Bold type indicates primary RS.

### 3.4. TPUS techniques

Technical details of TPUS procedures varied across studies. Table 2b summarizes the patient positioning, transducer orientation, and additional techniques to enhance the detection of fistulas, internal openings, and abscesses. Twenty-three studies^11–18^^,^^20^^,^^22^^,^^24^^,^^25^^,^^27–33^^,^^35–37^^,^^39^ described patient positioning for TPUS, with the most commonly employed position being left lateral (20 studies), followed by the supine lithotomy position (11 studies). The positions were not mutually exclusive, as some studies utilized both.

**Table 2b.** Characteristics of TPUS techniques performed.

Study	Year	Mode	Position for TPUS	Transducer placed over external opening	Placement of transducer	Additional Techniques
**Stewart** ^11^	2001	B-Mode	Supine lithotomy, left lateral	Yes	Longitudinal, oblique	–
**Bonatti** ^12^	2004	B-Mode and color Doppler	Left lateral	NA	Longitudinal	–
**Mallouhi** ^13^	2004	B-Mode and color Doppler	Left lateral	NA	Longitudinal, oblique	–
**Wedemeyer** ^14^	2004	B-Mode and color Doppler	Left lateral	Yes	Longitudinal, oblique, transverse	Patients to bear down
**Zbar** ^15^	2006	B-mode	Left lateral	Yes	Longitudinal, transverse	Use H_2_O_2_ to delineate fistula
**Domkundwar** ^16^	2007	B-Mode and color Doppler	Supine lithotomy, left lateral	NA	Longitudinal, oblique	–
**Kleinubing** ^35^	2007	B-mode	Supine lithotomy	NA	Longitudinal, oblique, transverse	Use H_2_O_2_ to delineate fistula
**Maconi** ^17^	2007	B-mode	Left lateral	Yes	Longitudinal, oblique, transverse	–
**Maconi** ^18^	2013	B-mode	Left lateral	Yes	Longitudinal, oblique, transverse	Translabial approach for anovulvular fistula
**Nevler** ^19^	2013	B-mode	NA	Yes	Longitudinal, oblique, transverse	–
**Plaikner** ^20^	2014	B-Mode and color Doppler	Left lateral	Yes	Longitudinal, oblique, transverse	–
**Terracciano** ^21^	2014	B-mode	NA	NA	NA	–
**Bor** ^23^	2016	B-mode	NA	NA	NA	–
**Terracciano** ^22^	2016	B-mode, color Doppler and power Doppler	Left lateral	NA	Longitudinal, transverse	–
**Puranik** ^24^	2017	B-mode	Supine lithotomy	Yes	Longitudinal, oblique, transverse	Bear down for air movement in fistula
**Fateh** ^25^	2017	B-mode	Left anterior oblique, flexed knees	NA	Longitudinal, transverse	–
**Lee** ^26^	2018	B-mode	NA	NA	NA	–
**Yan** ^27^	2018	B-mode	Supine lithotomy, left lateral	Yes	Longitudinal, transverse	Empty bowels
**Anand** ^28^	2022	B-mode	Supine lithotomy, left lateral	NA	Longitudinal, transverse	–
**Ding** ^29^	2022	B-mode	Supine lithotomy	NA	NA	–
**Jung** ^30^	2022	B-mode	Left lateral	NA	Longitudinal, transverse	–
**Boles** ^31^	2022	B-mode	Supine lithotomy, left lateral	NA	NA	Injection of saline for high tract fistula
**Singh** ^32^	2022	B-Mode and color Doppler	Supine lithotomy, left lateral	Yes	Longitudinal, oblique, transverse	Bear down for air movement in fistula
**Altam** ^33^	2023	B-Mode and color Doppler	Supine lithotomy, left lateral	Yes	Longitudinal, transverse	–
**Hosokawa** ^34^	2023	B-mode	NA	NA	Longitudinal, transverse	Use of oral analgesia if required
**Garg** ^36^	2023	B-mode	Left lateral and lithotomy positioning	NA	NA	–
**Yang** ^37^	2024	3D B-mode and SonoVue enhanced	Left lateral	NA	Longitudinal, transverse, sagittal, and 3D	Use SonoVue to delineate fistula. Gentle knead over perineal area after injection
**Islam** ^38^	2024	B-mode	NA	NA	NA	–
**Chang** ^39^	2025	B-mode	Left lateral	Yes	NA	–

Abbreviations: TPUS, transperineal ultrasound; NA, not available; H_2_O_2_, hydrogen peroxide.

Nine studies^12–14^^,^^16^^,^^22^^,^^32^^,^^33^^,^^36^^,^^39^ used Doppler ultrasound to aid in fistula characterization with two studies^16^^,^^22^ using it to distinguish between active and non-active fistulas, while one study^14^ used Doppler to differentiate between blood vessels and fistulas. Three studies^14^^,^^24^^,^^32^ recommended bearing down to enhance air movement within the fistula, thereby improving visualization of the fistulous tract. Several studies have explored injection of contrast agents for delineating the fistula tract, with two studies^15^^,^^35^ using hydrogen peroxide and one^37^ using SonoVue, while another^31^ used normal saline for high-tract fistulas. Additionally, one study^27^ recommended bowel emptying prior to TPUS, and another^34^ recommended the use of oral analgesia if necessary.

### 3.5. Diagnostic accuracy of TPUS for fistula detection

Of 27 studies^11–28^^,^^30–36^^,^^38^^,^^39^ that investigated FD, 18 studies^12–14^^,^^16–18^^,^^22–25^^,^^27^^,^^29–36^ defined a perianal fistula as the presence of a hypoechoic tract with or without air or fluid trapping while eight studies^11^^,^^14–16^^,^^19^^,^^27^^,^^32^^,^^33^ included sphincter defect assessment (Table S2).

**Figure 2.**
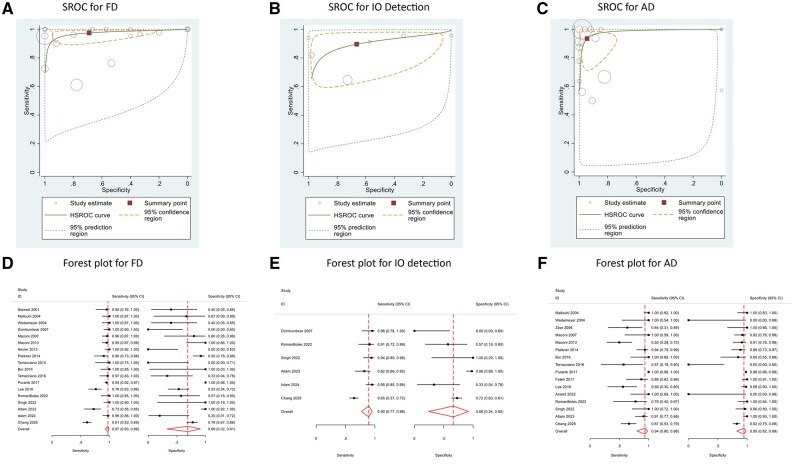
(A–C) Summary receiver operating curves (SROC) for fistula detection (FD), internal opening (IO), and abscess detection (AD). (D–F) Forest plots for FD, IO, and AD.

Eighteen studies^11^^,^^13^^,^^14^^,^^16–24^^,^^26^^,^^31–33^ qualified for meta-analysis (Figure 2D, Table 3), yielding a pooled sensitivity and specificity for FD of 97.5% (95% confidence interval [CI] 92.6, 99.2) and 69.0% (95% CI 32.8, 91.0) respectively with a diagnostic odds ratio (DOR) of 86.11 (95% CI 8.27, 405.84). Significant heterogeneity was observed (*I*2=99.0%, χ2=134.6; *P* < .001) and the accuracy of TPUS for FD was 88.0% (Figure 2A, Table 3).

**Table 3.** Analysis for diagnostic accuracy of TPUS for fistula detection, fistula classification and abscess detection.

			Meta-analytic summary estimates						
Category	Group	Number of patients	Sensitivity, % (95% CI)	Specificity, % (95% CI)	Positive LR (95% CI)	Negative LR (95% CI)	Diagnostic OR (95% CI)	AUC for SROC (95% CI)	Accuracy (%)
**Fistula detection**	Total	1474^a^	97.5 (92.6, 99.2)	69.0 (32.8, 91.0)	3.14 (1.11, 8.88)	0.04 (0.01, 0.10)	86.11 (18.27, 405.84)	0.97 (0.95, 0.98)	88.0
	CD	337^b^	96.2 (88.5, 98.8)	62.5 (29.9, 86.7)	2.57 (1.10, 6.00)	0.06 (0.02, 0.20)	42.68 (7.69, 236.89)	0.93 (0.91, 0.95)	86.4
	Adult	1369^c^	97.7 (93.1, 99.3)	75.8 (38.2, 94.1)	4.04 (1.19, 13.66)	0.03 (0.01, 0.09)	132.60 (26.80, 656.30)	0.98 (0.96, 0.99)	89.3
**Fistula classification**	Total	585^d^	80.3 (76.9, 83.4)						88.6
	CD	169^e^	87.6 (81.7, 91.8)						87.6
**IO detection**	Total	481^f^	89.6 (77.2, 95.7)	66.3 (24.1, 92.4)	2.66 (0.82, 8.65)	0.16 (0.07, 0.36)	17.00 (3.04, 95.10)	0.90 (0.87, 0.92)	77.8
**Abscess detection**	Total	1276^g^	93.5 (80.2, 98.1)	94.5 (82.4, 98.4)	17.00 (4.93, 58.70)	0.07 (0.02, 0.23)	247.90 (38.00, 1616.50)	0.98 (0.97, 0.99)	91.8
	CD	209^h^	85.5 (44.9, 97.7)	78.1 (28.5, 96.9)	3.90 (0.70, 21.70)	0.19 (0.03, 1.05)	21.00 (1.20, 366.40)	0.89 (0.86, 0.92)	83.3

a Number of studies: 18 ^11^^,^^13^^,^^14^^,^^16–24^^,^^26^^,^^31–33^^,^^38^^,^^39^.

b Number of studies: 8 ^11^^,^^14^^,^^17^^,^^18^^,^^21–23^^,^^26^.

c Number of studies: 16 ^11^^,^^13^^,^^14^^,^^16–20^^,^^22–24^^,^^31–33^^,^^38^^,^^39^.

d Number of studies: 11 ^11^^,^^15^^,^^17–19^^,^^22^^,^^25^^,^^33^^,^^36^^,^^37^^,^^39^.

e Number of studies: 5 ^11^^,^^17–19^^,^^22^.

f Number of studies: 6 ^16^^,^^31–33^^,^^38^^,^^39^.

g Number of studies: 16 ^13–15^^,^^17^^,^^18^^,^^20^^,^^22–26^^,^^28^^,^^31–33^^,^^39^.

h Number of studies: 6 ^14^^,^^17^^,^^18^^,^^22^^,^^23^^,^^26^.

Abbreviations: TPUS, transperineal ultrasound; CD, Crohn’s disease; IO, internal opening; OR, odd’s ratio; LR, likelihood ratio.

### 3.6. Diagnostic accuracy of TPUS for fistula classification and detection of internal opening

FC is discussed in 23 studies^11^^,^^13–20^^,^^22–30^^,^^32^^,^^33^^,^^36^^,^^37^^,^^39^, with Park’s classification being the most commonly used, followed by the American Gastroenterology Association (AGA) classification (Table S2). Eleven studies^11^^,^^15^^,^^17–19^^,^^22^^,^^25^^,^^33^^,^^36^^,^^37^^,^^39^ reported FC data using Park’s classification, yielding a pooled sensitivity of 80.3% (95% CI 76.9, 83.4) and accuracy of 88.6%. The FC data demonstrate moderate heterogeneity (*I*2=45.2%, χ^2^=12.8; *P* = .08). Sensitivity for FC was further stratified by fistula type, with a pooled sensitivity of 80.4%, 89.7%, 36.6%, and 38.9% for intersphincteric fistula (ISF), transphincteric fistula (TSF), suprasphincteric fistula (SSF), and extrasphincteric fistula (ESF) respectively.

IO was defined in eight studies^19^^,^^20^^,^^27^^,^^29^^,^^31^^,^^33^^,^^35^^,^^37^ using the Cho criteria, which was developed for endosonographic detection of IO of fistula.^40^ Six studies^16^^,^^31–33^^,^^38^^,^^39^ were included for IO detection meta-analysis (Figure 2E, Table 3) and the pooled sensitivity and specificity were 89.6% (95% CI 77.2, 95.7) and 66.3% (CI 95% 24.1, 92.4) respectively while the DOR was 17.00 (95% CI 3.04, 95.1). There was significant heterogeneity among studies for IO detection (*I*2=92.0%, χ^2^=25.1; *P* < .001). The accuracy of TPUS for the detection of IO was 77.8% (Figure 2B, Table 3).

### 3.7. Diagnostic accuracy of TPUS for abscess detection

Of 18 studies^13–15^^,^^17–20^^,^^22–26^^,^^28^^,^^31–34^^,^^39^ on abscess detection, ten^13^^,^^14^^,^^18^^,^^22–25^^,^^31^^,^^33^^,^^34^ defined an abscess as a hypoechoic to anechoic mass with material, gas, or positive compression sign. Fourteen studies^13–15^^,^^17–20^^,^^22–24^^,^^32–34^^,^^39^ investigated fistula–abscess connections and four studies^13^^,^^20^^,^^22^^,^^33^ utilized Doppler to aid abscess detection (Table S3).

Sixteen studies^13–15^^,^^17^^,^^18^^,^^20^^,^^22–26^^,^^28^^,^^31–33^^,^^39^ were suitable for meta-analysis (Figure 2F, Table 3) showing pooled sensitivity and specificity of 93.5% (CI 95% 80.2, 98.1) and 94.5% (CI 95% 82.4, 98.4) respectively. The pooled DOR was 247.9 (95% 38.0, 1616.5). There was significant heterogeneity among studies (*I*2=92.0%, χ^2^=24.0; *P* < .001). The accuracy of AD with TPUS was 91.8% (Figure 2C, Table 3).

### 3.8. Subgroup analysis

**Figure 3.**
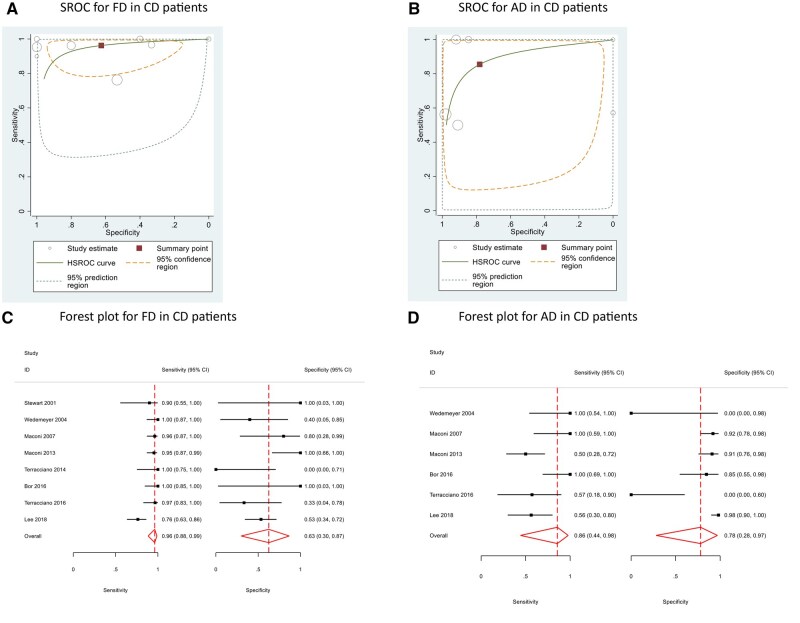
(A–B) Summary receiver operating curves (SROC) for fistula detection (FD) and abscess detection (AD) in CD patients. (C–D) Forest plots for FD and AD in CD patients.

Subgroup analysis on eight studies^11^^,^^14^^,^^17^^,^^18^^,^^21–23^^,^^26^ on CD patients described a pooled sensitivity and specificity of 96.2% (95% CI 88.5, 98.8) and 62.5% (95% CI 29.9, 86.7) respectively with a DOR and accuracy of 42.68 (95% CI 7.69, 236.89) and 86.4% for FD. The pooled sensitivity for FC and AD for CD patients was 87.6% (95% CI 81.7, 91.8) and 85.5% (95% CI 44.9, 97.7) respectively (Figure 3C, D, Table 3).

The diagnostic performance of TPUS was analyzed in comparison with MRI and EUA for FD and AD separately (Table S4). The pooled sensitivity of TPUS compared with MRI was 98.9% (95% CI 77.4, 99.9) for FD and 88.0% (95% CI 64.1, 96.8) for AD while comparing with EUA was 97.3% (95% CI 85.4, 99.6) for FD and 91.1% (95% CI 74.6, 97.2) for AD.

Sensitivity analyses showed an acceptable difference (±10% difference) for all primary domains tested except for specificity of FD (86.7% in sensitivity analyses vs 69.0% in primary analysis).

## 4. Discussion

This systematic review and meta-analysis provide an updated assessment of the diagnostic accuracy of TPUS for detecting perianal disease related to CD. Our results demonstrate that TPUS has a high diagnostic accuracy for FD, FC, IO detection, and AD, performing comparably to MRI, TRUS, and EUA.

Compared to the previously published meta-analysis by Maconi et al., our review demonstrates comparable pooled sensitivity for FD (97.5% vs 98.0%) and IO detection (89.6% vs 91.0%), higher pooled sensitivity for AD (93.5% vs 86.0%) but lower sensitivity for FC (80.3% vs 92.0%).^6^ The analysis of FC data was performed only on studies that reported classification based on Park’s classification because it is a widely used classification in studies and in surgical practice. Sensitivity of FC is reduced for high fistulas (such as suprasphincteric and extrasphincteric), probably reflecting the limitation of TPUS probes, which allow for detailed study of the superficial perineal layer at the expense of image resolution at deeper layers.^22^ In contrast, MRI does not share this limitation due to its consistency with superior soft-tissue contrast regardless of fistula depth.

In the management of perianal fistulas, the presence of proctitis, the fistula classification (in relation to the external sphincter), the number and location of internal openings, and the activity of the fistula tract play a crucial role in deciding for the best treatment option. Among the publications reviewed, fistula count, classification type, and presence of abscesses were generally included. The presence of proctitis was not included as a study or reporting criterion in any of the reviewed studies. This may be due to the previous lack of correlation between bowel wall thickness (BWT) and the definition of proctitis, though recent studies have identified a rectal BWT of 4 mm as indicative of inflammation.^41^ A recent collaborative review by leading European societies in IBD and gastrointestinal radiology proposed a standardized reporting template for the imaging of pfCD, applicable to both MRI and TPUS.^42^

While TPUS generally requires minimal preparation, various techniques have been explored to improve visualization of fistula tracts or abscesses. Among these, the injection of contrast agents directly into the fistula tract has been investigated in a limited number of studies. These contrast agents, in particular hydrogen peroxide and SonoVue, improve visualization by creating numerous hyperechogenic interfaces that delineate fistula tracts. However, outcomes have been variable. Two studies (*n* = 63) investigated the use of hydrogen peroxide with TPUS and found no overall improvement although it facilitated rectovaginal fistula tracking in two patients.^15^^,^^35^ More recently, injection of SonoVue contrast into fistula tracts has been shown to significantly improve accuracy of complex fistula classification (98.3% vs 85.0%), detection of fistula branches (92.6% vs 70.4%), and IO (97.1% vs 80.9%), although it did not significantly increases accuracy in FC (96.7% vs 95.0%).^37^ A comparative study using TRUS showed similar accuracy between hydrogen peroxide and SonoVue in detecting IO and outperformed non-contrast scans.^43^ Although these contrasts are generally safe, hydrogen peroxide can cause a transient local burning sensation around the perineal region or irritation to rectal mucosa whereas SonoVue has been associated with significantly less patient-reported pain.^35^^,^^43^^,^^44^ Moreover, image quality and accuracy of FD are enhanced with SonoVue due to the uniform distribution and sustained stability of microbubbles within the fistula tracts.^37^^,^^43^ Besides hydrogen peroxide and SonoVue, methods such as saline injection into the fistula tract and bearing down to induce air movement in the fistula have been described, though no comparative studies have evaluated their effectiveness in improving FD or FC.

TPUS showed lower accuracy for FD and AD in CD patients than in the overall population, probably due to the greater complexity of CD-related fistulas.^4^ However, the true incidence of pfCD in this meta-analysis is likely to be underestimated, as most studies do not specify the perianal fistula etiology. Furthermore, only one study on FD with a reference standard (*n* = 26) included CD and non-CD patients, limiting conclusions.^11^

This meta-analysis highlights the utility of TPUS as a valuable, highly accurate tool for diagnosing and assessing perianal conditions, particularly in patients with a suspected perianal abscess. TPUS has high diagnostic accuracy for AD, underscoring its importance in the initial evaluations of these patients. Given that 30%-70% of patients with perianal abscesses may present with a concomitant perianal fistula,^45^ practitioners are encouraged to thoroughly evaluate fistula presence during TPUS assessments. Moreover, TPUS has potential for monitoring perianal fistulas following treatment, though only one study has addressed this application.^30^ Delineating the different stages of healing in pfCD remains an unmet clinical need. While TPUS may serve as a practical first-line tool due to its accessibility and safety profile, initial MRI remains essential for comprehensive perianal fistula mapping prior to definitive management. Thereafter, TPUS could potentially be used for more frequent, non-invasive monitoring. Further research is warranted to establish standardized protocols for TPUS in the longitudinal management of perianal fistulas. This should establish its potential capacity to differentiate between fluid, granulation tissue, and fibrosis of the fistula tract. Additionally, future TPUS studies could explore its potential for differentiating between cryptoglandular and CD-related fistulas. This differentiation may be achieved by assessing specific features such as the presence of fistula debris, bifurcation, and associated rectal inflammation and could be further supported by deep learning models, as seen in recent MRI studies.^46^^,^^47^

Our systematic review has several limitations. First, the number of studies reporting the diagnostic accuracy of TPUS against a reference standard is small. Although we used broad search terms to increase the yield of available studies, the available evidence was of low quality, underscoring the need for further high-quality research in this area. Second, selection bias may occur due to the focus on patients with perianal complaints, potentially leading to higher sensitivities for FD and AD. Third, significant heterogeneity was observed due to variations in reference standards, procedural timing, imaging techniques, outcome definitions, data capture methods (per-patient vs per-lesion reporting), and patient demographics. To improve on consistency of reference standards, studies that included CT as part of a composite reference standards were removed from the meta-analysis.^12^^,^^34^ Additionally, a bivariate random-effects model was used to account for residual between-study heterogeneity and the correlation between sensitivity and specificity within and across studies. Finally, TPUS is operator-dependent, with its reliability influenced by the operator’s experience. The absence of operator experience data in many studies is a significant limitation of this review. Future research focused on assessing reliability and interobserver agreement is essential to address these limitations.

In conclusion, TPUS is a highly accurate, non-invasive tool for detecting perianal fistulas and abscesses in both CD and non-CD patients, supporting its role as a first-line diagnostic tool. Further research should investigate the utility of TPUS in the monitoring of perianal fistulas to better define its role in monitoring patients with pfCD.

## Supplementary Material

jjag032_Supplementary_Data

## Data Availability

Data, analytic methods, and study materials will be made available to other researchers on reasonable request.
