# Supplementary material for: Transperineal ultrasonography in detecting penetrating perianal disease: a systematic review and meta-analysis
Source: J Crohns Colitis. 2026 Mar 24;20(3):jjag032. doi: 10.1093/ecco-jcc/jjag032 (PMC13010342; doi:10.1093/ecco-jcc/jjag032)
Supplement: jjag032_Supplementary_Data [file jjag032_supplementary_data.zip › Supplementary Files 1 (PROSPERO).pdf]

A systematic review of the accuracy of transperineal ultrasound in the detection of perianal fistulas

Chong Teik Lim, Maarten Pruijt

To enable PROSPERO to focus on COVID-19 submissions, this registration record has undergone basic automated checks for eligibility and is published exactly as submitted. PROSPERO has never provided peer review, and usual checking by the PROSPERO team does not endorse content. Therefore, automatically published records should be treated as any other PROSPERO registration. Further detail is provided here.

Citation

Chong Teik Lim, Maarten Pruijt. A systematic review of the accuracy of transperineal ultrasound in the detection of perianal fistulas. PROSPERO 2024 CRD42024511822. Available from https://www.crd.york.ac.uk/PROSPERO/view/CRD42024511822.

PREVIEW

REVIEW TITLE AND BASIC DETAILS

Review title

A systematic review of the accuracy of transperineal ultrasound in the detection of perianal fistulas

Review objectives

What is the accuracy of transperineal ultrasonography in detecting perianal fistula compared to MRI, transrectal endoscopic ultrasound, and/or examination under anaesthesia?

SEARCHING AND SCREENING

Searches

Search strategies were focused on journals with topics regarding transperineal ultrasonography and perianal fistula.Search was conducted in these databases:MEDLINE (ovid) 1945 till February 4th 2025EMBASE (ovid) 1974 till February 4th 2025

Study design

Inclusion criteria:

• Full text peer-reviewed articles

• Case series including ≥10 patients

• Observational studies (cross-sectional, longitudinal, case-control and cohort studies)

• Conference abstracts published from 1st January 2021

Exclusion criteria:

• Systematic reviews and meta-analyses

• Review articles, editorials, letters and commentaries

• Studies with insufficient data to determine sensitivity, specificity, PPV or NPV calculations

• Case reports and case series with <10 patients

• Conference abstracts published before 1st January 2021

• In vitro, ex vivo and animal studies

• Published protocols without original data

ELIGIBILITY CRITERIA

Condition or domain being studied
Perianal Fistula. Inflammatory bowel disease.

Population
Pediatrics and adult populations with confirmed or suspected diagnosis of perianal fistula.

Intervention(s) or exposure(s)
Use of transperineal ultrasonography for diagnosis of perianal fistula

Comparator(s) or control(s)
Use of Examination under Anasthesia, MRI and/or transrectal endoscopic ultrasonography for diagnosis of perianal fistula.

OUTCOMES TO BE ANALYSED

Main outcomes
To examine the accuracy of transperineal ultrasonography in detecting, characterising and classifying perianal fistula compared to MRI, transrectal endoscopic ultrasound, and/or examination under anaesthesia.

Measures of effect

Additional outcomes
To assess whether ultrasound modalities like B-mode, contrast enhanced transperineal ultrasonography with hydrogen peroxide or Sonovue, elastography and Doppler increase the diagnostic yield in detecting perianal fistulas.
To examine the accuracy of transperineal ultrasonography in detecting, characterising and classifying perianal fistula in patients with Crohn's disease compared to MRI, transrectal endoscopic ultrasound, and/or examination under anaesthesia.
To compare the accuracy of transperineal ultrasonography in detecting, characterising and classifying perianal fistula in paediatrics populations to adult populations.

DATA COLLECTION PROCESS

Data extraction (selection and coding)
Data will be extracted independently by two reviewers and entered into Excel spreadsheets (Microsoft Inc., Redmond, Washington, USA). The following data factors will be extracted from each study: First author, year of publication, study design, country, number of participants, age, gender, probe used, number of operators, ultrasonographic device, type of exam (B-mode, CEUS, Elastography and/or Doppler), additional methods used to increase yield of investigation (if any), number of patients with perianal fistula, types of perianal fistula (classified in accordance to Park's classification, St James Hospital's classification or American Gastroenterology Association), location of internal of perianal fistula, perianal abscess, stricture/stenosis (length), presence of rectovaginal fistula, sensitivity, specificity, PPV, NPV, true positive, false negative, false positive, true negative, lag time between transperineal ultrasound and EUA/MRI/transrectal endoscopic ultrasound.

Risk of bias (quality) assessment
Quality and risk of bias are assessed independently by two reviewers (CTL and MP) using an adaption of the QUADAS-2 tool. Any discrepancies on the eligibility of studies to be included will be resolved through discussion or resolved through a third reviewer. In systematic reviews comparing an index test and a reference test, the QUANDAS-2 tool is used to determine the quality of each individual study.

PLANNED DATA SYNTHESIS

Strategy for data synthesis
We expect to include studies with heterogeneous data.Therefore, a descriptive data synthesis will be conducted.

Analysis of subgroups or subsets
The secondary outcomes / subgroup analysis planned:
a. Comparing different ultrasound modalities on diagnostic yield. (contrast enhanced transperineal ultrasonography with hydrogen peroxide or Sonovue, elastography and Doppler)
b. Accuracy of Transperineal ultrasonography in patients' with Crohn's disease diagnosis.
c. Compare accuracy of transperineal ultrasonography in pediatrics vs adult popultation (>18 years old)

REVIEW AFFILIATION, FUNDING AND PEER REVIEW

Review team members
Dr Chong Teik Lim (review guarantor and contact) Singapore General Hospital. Singapore.

No conflict of interest declared.

Dr Maarten Pruijt. Amsterdam University Medical Centre. Netherlands.

No conflict of interest declared.

Named contact
Dr Chong Teik Lim (thomson.lim.c.t@singhealth.com.sg). Singapore General Hospital. Singapore.

Review affiliation
Amsterdam University Medical Centre

Funding source
No funding.

TIMELINE OF THE REVIEW

Review timeline
Start date: 2 January 2024. End date: 31 May 2025.

Date of first submission to PROSPERO
10 February 2024

Date of registration in PROSPERO
21 February 2024

CURRENT REVIEW STAGE

Publication of review results
The intention is not to publish the review once completed.

Stage of the review at this submission

Review stage	Started	Completed
Pilot work	✓	✓
Formal searching/study identification	✓	✓
Screening search results against inclusion criteria	✓	✓
Data extraction or receipt of IPD	✓	✓
Risk of bias/quality assessment	✓	✓
Data synthesis	✓	✓

Review status
The review is completed.

ADDITIONAL INFORMATION

PROSPERO version history

- Version 1.4, published 05 Nov 2024
- Version 1.3, published 21 Sep 2024
- Version 1.2, published 21 Mar 2024
- Version 1.1, published 21 Feb 2024
- Version 1.0, published 21 Feb 2024

Review conflict of interest

None known

Country

Netherlands, Singapore

Medical Subject Headings

Anesthesia; Endosonography; Humans; Magnetic Resonance Imaging; Rectal Fistula; Ultrasonography

Revision note

Updated Search up till February 2025.

Disclaimer

The content of this record displays the information provided by the review team. PROSPERO does not peer review registration records or endorse their content.

PROSPERO accepts and posts the information provided in good faith; responsibility for record content rests with the review team. The guarantor for this record has affirmed that the information provided is truthful and that they understand that deliberate provision of inaccurate information may be construed as scientific misconduct.

PROSPERO does not accept any liability for the content provided in this record or for its use. Readers use the information provided in this record at their own risk.

Any enquiries about the record should be referred to the named review contact
