# Supplementary material for: Transperineal ultrasonography in detecting penetrating perianal disease: a systematic review and meta-analysis
Source: J Crohns Colitis. 2026 Mar 24;20(3):jjag032. doi: 10.1093/ecco-jcc/jjag032 (PMC13010342; doi:10.1093/ecco-jcc/jjag032)
Supplement: jjag032_Supplementary_Data [file jjag032_supplementary_data.zip › Supplementary Files 2 (Search Strategy).docx]

What is the accuracy of transperineal ultrasonography in detecting perianal fistula compared to MRI, transrectal endoscopic ultrasound, and/or examination under anaesthesia?

Lim Chong Teik

Search: Faridi Jamaludin, Amsterdam UMC location University of Amsterdam, Medical Library AMC, Meibergdreef 9, Amsterdam, The Netherlands

UPDATE SEARCH:

4-2-2025:

| Databases: | Before deduplication | After deduplication |
| --- | --- | --- |
| Medline (Ovid), Embase (Ovid) |  |  |
| Total | 1541 | 1059 |

*Lobbestael, G. (2023). DedupEndNote (Version 1.0.0) [Computer software]. https://github.com/globbestael/DedupEndNote

MEDLINE (OVID):

Database(s): Ovid MEDLINE(R) Epub Ahead of Print and In-Process, In-Data-Review & Other Non-Indexed Citations and Daily February 03, 2025
Search Strategy:

| # | Searches | Results |
| --- | --- | --- |
| 1 | (transperineal* or perineal* or perianal*).ti,ab,kf. | 30864 |
| 2 | exp Fistula/ or (fistulae or fistula* or abscesses).ti,ab,kf. | 165469 |
| 3 | exp ultrasonography/ | 506289 |
| 4 | (ultraso* or sonograph*).ti,ab,kf. | 539607 |
| 5 | 3 or 4 | 794096 |
| 6 | 1 and 2 and 5 | 487 |
| 7 | limit 6 to yr="2023 -Current" | 511 |

Embase (OVID):

Database(s): Embase Classic+Embase 1947 to 2025 February 03
Search Strategy:

| # | Searches | Results |
| --- | --- | --- |
| 1 | (transperineal* or perineal* or perianal*).ti,ab,kf. | 51949 |
| 2 | exp fistula/ or (fistulae or fistula* or abscesses).ti,ab,kf. | 277439 |
| 3 | exp echography/ | 1134663 |
| 4 | (ultraso* or sonograph*).ti,ab,kf. | 796339 |
| 5 | 3 or 4 | 1506290 |
| 6 | 1 and 2 and 5 | 1035 |
| 7 | limit 6 to yr="2023 -Current" | 1030 |
