# Supplementary material for: Transperineal ultrasonography in detecting penetrating perianal disease: a systematic review and meta-analysis
Source: J Crohns Colitis. 2026 Mar 24;20(3):jjag032. doi: 10.1093/ecco-jcc/jjag032 (PMC13010342; doi:10.1093/ecco-jcc/jjag032)
Supplement: jjag032_Supplementary_Data [file jjag032_supplementary_data.zip › Supplementary Table 1.docx]

| **Study** | **RISK OF BIAS** | | | | **APPLICABILITY CONCERNS** | | |
| --- | --- | --- | --- | --- | --- | --- | --- |
|  | **PATIENT SELECTION** | **INDEX TEST** | **REFERENCE STANDARD** | **FLOW AND TIMING** | **PATIENT SELECTION** | **INDEX TEST** | **REFERENCE STANDARD** |
| Stewart 2001 | ☺ | ☺ | ? | ? | ☺ | ? | ? |
| Bonatti 2004 | ? | ☺ | ? | ? | ? | ☺ | ☹ |
| Mallouhi 2004 | ☺ | ☺ | ? | ☺ | ? | ☺ | ? |
| Wedemeyer 2004 | ☺ | ☺ | ☺ | ☺ | ☺ | ☺ | ☺ |
| Zbar 2006 | ? | ☺ | ☹ | ☺ | ☹ | ☺ | ? |
| Domkundwar 2007 | ☹ | ☺ | ☹ | ? | ☹ | ☺ | ☹ |
| Kleinubing 2007 | ? | ☺ | ☺ | ? | ☹ | ☺ | ☺ |
| Maconi 2007 | ☺ | ☺ | ☺ | ☺ | ☺ | ☺ | ☺ |
| Maconi 2013 | ☺ | ☺ | ☺ | ☺ | ☺ | ☺ | ☺ |
| Nevler 2013 | ☺ | ☺ | ? | ☺ | ☺ | ☺ | ☺ |
| Plaikner 2014 | ☺ | ☺ | ☺ | ☺ | ☹ | ☺ | ☺ |
| Terracciano 2014 | ☺ | ☺ | ? | ☺ | ☺ | ☺ | ? |
| Bor 2016 | ☹ | ☺ | ☺ | ☺ | ☺ | ☺ | ☺ |
| Terracciano 2016 | ☺ | ☺ | ☺ | ☺ | ☺ | ☺ | ☺ |
| Puranik 2017 | ? | ☺ | ☹ | ? | ☹ | ☺ | ☹ |
| Fateh 2017 | ☹ | ☺ | ☹ | ? | ☹ | ☺ | ☺ |
| Lee 2018 | ☹ | ☺ | ? | ☺ | ☺ | ☺ | ? |
| Yan 2018 | ☹ | ☺ | ? | ? | ? | ☺ | ☺ |
| Anand 2022 | ☹ | ☺ | ? | ? | ? | ☺ | ☺ |
| Ding 2022 | ☺ | ☹ | ☹ | ? | ☹ | ? | ? |
| Jung 2022 | ? | ☺ | ☺ | ☺ | ☺ | ☺ | ☺ |
| Boles 2022 | ? | ☺ | ? | ☺ | ☹ | ☺ | ☺ |
| Singh 2022 | ☹ | ☺ | ? | ? | ? | ☺ | ☺ |
| Altam 2023 | ☹ | ☺ | ☹ | ☺ | ? | ☺ | ☺ |
| Hosokawa 2023 | ? | ☺ | ? | ? | ? | ☺ | ☹ |
| Garg 2023 | ? | ☺ | ☺ | ? | ☺ | ☺ | ☺ |
| Yang 2024 | ☹ | ☺ | ? | ☺ | ☹ | ☺ | ☺ |
| Islam 2024 | ? | ? | ? | ? | ☺ | ☺ | ☺ |
| Chang 2025 | ? | ☺ | ☺ | ? | ? | ☺ | ☺ |
