# Supplementary material for: Transperineal ultrasonography in detecting penetrating perianal disease: a systematic review and meta-analysis
Source: J Crohns Colitis. 2026 Mar 24;20(3):jjag032. doi: 10.1093/ecco-jcc/jjag032 (PMC13010342; doi:10.1093/ecco-jcc/jjag032)
Supplement: jjag032_Supplementary_Data [file jjag032_supplementary_data.zip › Supplementary Table 2.docx]

| Study | Year | Fistula Classification | Hypoechoic Tract ± Fluid / Air Trapping | IO as defined by Cho criteria^40^ | Attempts to look for Sphincter Defect | Use of Doppler for fistula characterization |
| --- | --- | --- | --- | --- | --- | --- |
| Stewart^11^ | 2001 | Park’s | NA | NA | Yes | NA |
| Bonatti^12^ | 2004 | NA | Yes | NA | NA | Yes |
| Mallouhi^13^ | 2004 | Park’s | Yes | NA | NA | Yes |
| Wedemeyer^14^ | 2004 | Park’s | Yes | NA | Yes | Yes – differentiate from blood vessel |
| Zbar^15^ | 2006 | Park’s | NA | NA | Yes | NA |
| Domkundwar^16^ | 2007 | Park’s | Yes | NA | Yes | Yes (active vs non active) |
| Kleinubing^35^ | 2007 | NA | Yes | Yes | NA | NA |
| Maconi^17^ | 2007 | Park’s | Yes | NA | NA | NA |
| Maconi^18^ | 2013 | Park’s and AGA | Yes | NA | NA | NA |
| Nevler^19^ | 2013 | Park’s | NA | Yes | Yes | NA |
| Plaikner^20^ | 2014 | Park’s | NA | Yes | NA | NA |
| Terracciano^21^ | 2014 | NA | NA | NA | NA | NA |
| Bor^23^ | 2016 | AGA | Yes | NA | NA | NA |
| Terracciano^22^ | 2016 | Park’s and AGA | Yes | NA | NA | Yes (active vs non active) |
| Puranik^24^ | 2017 | Park’s | Yes | NA | NA | NA |
| Fateh^25^ | 2017 | St James | Yes | NA | NA | NA |
| Lee^26^ | 2018 | Park’s and St James | NA | NA | NA | NA |
| Yan^27^ | 2018 | AGA | Yes | Yes | Yes | NA |
| Anand^28^ | 2022 | Park’s and St James | NA | NA | NA | NA |
| Ding^29^ | 2022 | AGA | Yes | Yes | NA | NA |
| Jung^30^ | 2022 | Park’s and St James | Yes | NA | NA | NA |
| Boles^31^ | 2022 | NA | Yes | Yes | NA | NA |
| Singh^32^ | 2022 | Park’s | Yes | NA | Yes | Yes |
| Altam^33^ | 2023 | Park’s | Yes | Yes | Yes | Yes |
| Hosokawa^34^ | 2023 | NA | Yes | NA | NA | NA |
| Garg^36^ | 2023 | Park’s and St James | Yes | NA | NA | Yes |
| Yang^37^ | 2024 | Park’s | Yes | Yes | NA | NA |
| Islam^38^ | 2024 | NA | NA | NA | NA | NA |
| Chang^39^ | 2025 | Park’s | NA | NA | NA | Yes |
