# Supplementary material for: Transperineal ultrasonography in detecting penetrating perianal disease: a systematic review and meta-analysis
Source: J Crohns Colitis. 2026 Mar 24;20(3):jjag032. doi: 10.1093/ecco-jcc/jjag032 (PMC13010342; doi:10.1093/ecco-jcc/jjag032)
Supplement: jjag032_Supplementary_Data [file jjag032_supplementary_data.zip › Supplementary Table 3.docx]

| Study | Year | Description of hypoechoic to anechoic mass with material, gas or positive compression sign | Demonstration of connection with fistula | Use of doppler to help with abscess detection | Classification of abscess to superficial, deep or horseshoe |
| --- | --- | --- | --- | --- | --- |
| Mallouhi^13^ | 2004 | Yes | Yes | Yes | Yes |
| Wedemeyer^14^ | 2004 | Yes | Yes | NA | NA |
| Zbar^15^ | 2006 | NA | Yes | No | NA |
| Maconi^17^ | 2007 | NA | Yes | NA | Yes |
| Maconi^18^ | 2013 | Yes | Yes | NA | NA |
| Nevler^19^ | 2013 | NA | Yes | NA | Yes |
| Plaikner^20^ | 2014 | NA | Yes | Yes | Yes |
| Bor^23^ | 2016 | Yes | Yes | NA | NA |
| Terracciano^22^ | 2016 | Yes | Yes | Yes | Yes |
| Puranik^24^ | 2017 | Yes | Yes | No | NA |
| Fateh^25^ | 2017 | Yes | NA | NA | NA |
| Lee^26^ | 2018 | NA | NA | NA | NA |
| Anand^28^ | 2022 | NA | NA | NA | NA |
| Boles^31^ | 2022 | Yes | NA | NA | Yes |
| Singh^32^ | 2022 | NA | Yes | NA | NA |
| Altam^33^ | 2023 | Yes | Yes | Yes | NA |
| Hosokawa^34^ | 2023 | Yes | Yes | NA | NA |
| Chang^39^ | 2025 | NA | Yes | NA | Yes |
