# Supplementary material for: Transperineal ultrasonography in detecting penetrating perianal disease: a systematic review and meta-analysis
Source: J Crohns Colitis. 2026 Mar 24;20(3):jjag032. doi: 10.1093/ecco-jcc/jjag032 (PMC13010342; doi:10.1093/ecco-jcc/jjag032)
Supplement: jjag032_Supplementary_Data [file jjag032_supplementary_data.zip › Supplementary Table 4.docx]

|  | | | Meta-analytic summary estimates | | | | | | |
| --- | --- | --- | --- | --- | --- | --- | --- | --- | --- |
| Category | Group | Number of patients | Sensitivity, % (95% CI) | Specificity, % (95% CI) | Positive LR (95% CI) | Negative LR (95% CI) | Diagnostic OR (95% CI) | AUC for SROC (95% CI) | Accuracy (%) |
| Fistula Detection | MRI | 223^a^ | 98.9 (77.4, 99.9) | 52.1 (35.9, 67.9) | 2.07 (1.46, 2.93) | 0.02 (0.001, 0.55) | 94.30 (3.38, 2627.10) | 0.54 (0.49, 0.58) | 82.5 |
|  | EUA | 612^b^ | 97.3 (85.4, 99.6) | 52.8 (13.3, 89.1) | 2.06 (0.74, 5.75) | 0.05 (0.01, 0.22) | 40.60 (6.69, 264.94) | 0.95 (0.92, 0.96) | 80.9 |
| Abscess Detection | MRI | 221^c^ | 88.0 (64.1, 96.8) | 80.3 (1.6, 99.9) | 4.46 (0.06, 360.30) | 0.15 (0.03, 0.69) | 29.90 (0.12, 7179.30) | 0.90 (0.87, 0.92) | 90.0 |
|  | EUA | 464^d^ | 91.1 (74.6, 97.2) | 93.8 (84.0, 97.8) | 14.7 (5.08, 42.46) | 0.10 (0.03, 0.31) | 154.10 (20.1, 1183.20) | 0.98 (0.96, 0.99) | 87.1 |
