# Supplementary figures and images for: Transperineal ultrasonography in detecting penetrating perianal disease: a systematic review and meta-analysis
Source: J Crohns Colitis. 2026 Mar 24;20(3):jjag032. doi: 10.1093/ecco-jcc/jjag032 (PMC13010342; doi:10.1093/ecco-jcc/jjag032)

QUADAS-2 Domain

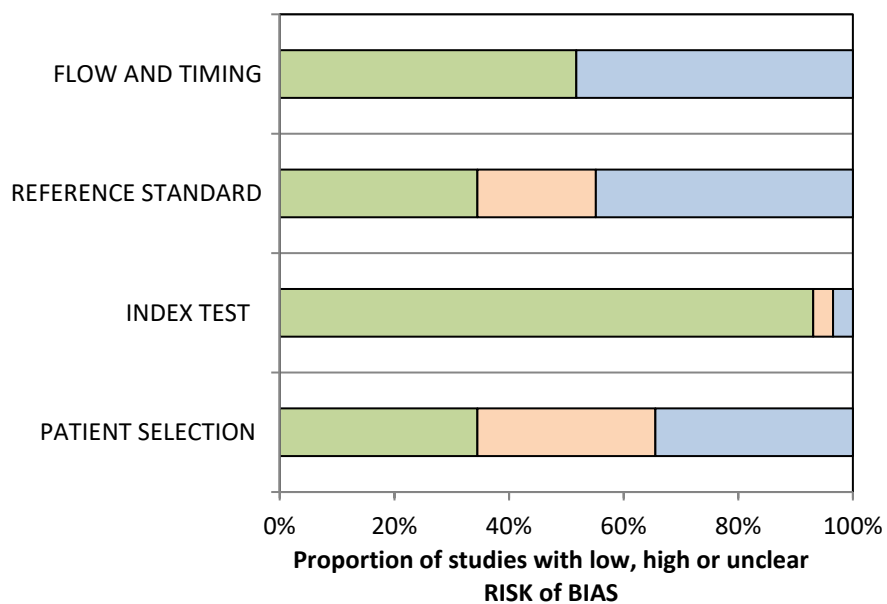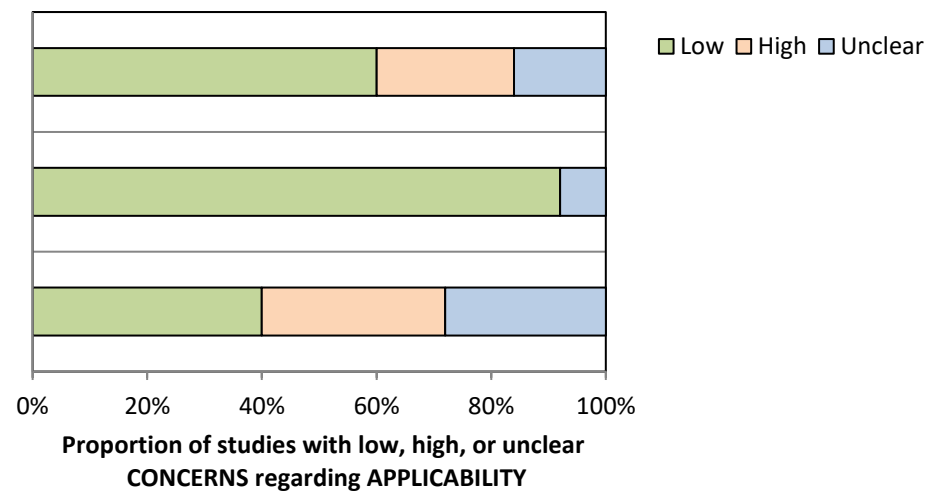

Supplement: jjag032_Supplementary_Data [file jjag032_supplementary_data.zip › Supplementary_Figure_1.pdf]
